# Commentary: Gray Matter Structural Alterations in Social Anxiety Disorder: A Voxel-Based Meta-Analysis

**DOI:** 10.3389/fpsyt.2019.00001

**Published:** 2019-01-22

**Authors:** Janna Marie Bas-Hoogendam

**Affiliations:** ^1^Institute of Psychology, Leiden University, Leiden, Netherlands; ^2^Department of Psychiatry, Leiden University Medical Center, Leiden, Netherlands; ^3^Leiden Institute for Brain and Cognition, Leiden, Netherlands

**Keywords:** social anxiety disorder, structural magnetic resonance imaging, gray matter volume, meta-analysis, voxel-based morphometry

## Introduction

Social anxiety disorder (SAD), an impairing and often chronic psychiatric disorder (1), has a lifetime prevalence between 6 and 13% (2–5) and is prevailing worldwide (6). At present, treatment for SAD is often suboptimal (7–10). Insight in the neurobiological changes underlying the socially-anxious brain is of utmost importance to improve preventive and therapeutic interventions.

Until now, several studies have examined alterations in brain structure associated with SAD, by using magnetic resonance imaging (MRI). This method enables investigating changes in gray matter (GM) (11). Results of MRI studies on GM characteristics related to SAD show, however, little consistency and have small effect sizes (12–14).

Recently, Wang et al. (15) described a voxel-based meta-analysis on GM volume (GMV) differences between SAD-patients and healthy participants. Such a meta-analytic review is very welcome in order to quantitatively summarize the results of previously published studies and to further increase our understanding of SAD-related GMV alterations. Unfortunately, the paper did not live up to its promise. Wang et al. state that SAD is associated with *increased* cortical and *decreased* subcortical GMVs, but these conclusions cannot be deduced from their data. Here, we want to point out several shortcomings that seriously affect this work.

## Methodological Issues

To start, several methodological details limit the authors' conclusions. First, the description of the analytical approach lacks clarity. According to the methods, “peak coordinates data of GMV differences found significant at the whole-brain level” were used to create maps of the effect size of GMV differences for each study, but three studies included in the meta-analysis did not report significant group differences at the whole-brain level (16–18) and two reported effects at an uncorrected significance level only (19, 20). Furthermore, the authors did not explain how the sensitivity analysis, discarding one study at a time, handles studies reporting null-effects.

Another methodological issue concerns the subgroup analysis on adult SAD-patients. This analysis includes 384 patients (all >18 years according to the methods, or ≥18 years according to the abstract), while the main analysis contained 480 patients. As the authors do not provide an overview of the samples which were excluded, we examined the study characteristics [Table 1 of the original article by Wang et al. (15)] and compared the number of participants. We assume that studies for which data on the age-range were, according to the authors, not available, were excluded (18, 21, 22). However, when reading the original papers, it seems very likely that these excluded studies also concern adult SAD-patients. This means that the different results of the subgroup analysis, compared to the main analysis, are most likely caused by changes in statistical power and cannot be attributed to age-effects. Future studies are needed to investigate the effect of age on SAD-related GMV alterations.

## Discussion of Results

Next, we feel the findings deserve more nuanced attention than the authors currently provide in the Discussion. In addition, findings of previous studies are not reflected accurately. We highlight two examples.

The first concerns the discussion of the precuneus results. The authors link their results of increased GMV in the left precuneus to the findings of “increased cortical thickness of the precuneus” by Brühl et al. (23) and Syal et al. (24). However, Syal and colleagues actually reported a thinning of the left precuneus related to SAD, implying decreased GMV.

Another example involves the authors' argumentation with respect to the GMV reduction in the left putamen. The authors argue that the age-related reduction in putamen volume in SAD-patients, reported by Potts et al. (25), is in line with their finding. We don't agree with this statement: Potts and colleagues indicate that there is “no statistically significant difference between social phobia patients and normal control subjects”; the “age-related reduction in putamen volumes in patients with social phobia that was greater than that seen in controls” (25) cannot be equated with the group difference reported in the present meta-analysis. Furthermore, it should be noted that recent work on putamen volume in SAD, which was probably not yet available at the time the meta-analysis was performed, implies changes in the opposite direction, namely increased GMV in the dorsal striatum in SAD (14, 16); these findings are supported by a positive relationship between social anxiety and GMV in the putamen in healthy women (26) and a positive correlation between the concept “intolerance of uncertainty” and putamen volume (27).

## Misrepresentation of Previous studies

The authors repeatedly miss the chance to summarize the details of the studies included in the meta-analysis in an insightful and correct way. For example, Table 1 (15) indicates that the paper describing a mega-analysis on the largest database of SAD structural MRI scans to date (16) and the paper by Irle et al. (19) do not provide scores on the Liebowitz Social Anxiety Scale (LSAS); these scores are, however, available for the majority of the SAD-patients (148/174) included in the mega-analysis (16), while the paper by Irle et al. reported scores on the two dimension scales of the LSAS (19). Furthermore, the authors inaccurately indicate that the paper by Meng et al. (28) does not provide the age of onset of SAD, while the number of SAD-patients taking medication in the study by Månsson et al. (20) is incorrectly reproduced.

Next, the main text of the paper does not always provide a comprehensive overview of ongoing research in SAD. For example, the authors describe that “only a few studies of SAD have explicitly controlled for depression comorbidity,” referring to three papers which are not part of the present meta-analysis (23, 24, 29). This is a valid point. However, the authors fail to mention that, given the high comorbidity rate between depression and SAD (30), completely excluding SAD-patients with comorbid depression could lead to biased results, as this could implicate that severe SAD-cases are excluded. In order to account for this comorbidity, sensitivity analyses, such as described by Bas-Hoogendam et al. (16) and Irle et al. (19), provide valuable information and deserve to be mentioned.

## Conclusion and Recommendations

To conclude, the results presented by Wang et al. need to be interpreted with caution. On the basis of the present meta-analysis, it is premature to conclude that SAD is associated with increased cortical and decreased subcortical GMVs. Meta-analyses on larger samples (31–33) are needed to improve our understanding of the SAD-related structural brain alterations.

## Author Contributions

The author confirms being the sole contributor of this work and has approved it for publication.

### Conflict of Interest Statement

The author declares that the research was conducted in the absence of any commercial or financial relationships that could be construed as a potential conflict of interest.
